# Penicillin G Benzathine management in Primary Health Care: nursing’s perspective

**DOI:** 10.1590/0034-7167-2024-0385

**Published:** 2026-05-11

**Authors:** Giovanna Martins Biondi, Daniela Sanches Couto, Lívia Cristina Scalon da Costa Perinoti, Tatiane Garcia do Carmo Flausino, Ana Joice de Barros Figueiredo, Rosely Moralez de Figueiredo

**Affiliations:** I Universidade Federal de São Carlos. São Carlos, São Paulo, Brazil; II Centro Universitário das Faculdades Associadas de Ensino. São João da Boa Vista, São Paulo, Brazil; III Universidade Federal de São Carlos, Hospital Universitário. São Carlos, São Paulo, Brazil; IV Universidade de Araraquara. Araraquara, São Paulo, Brazil

**Keywords:** Penicillin G Benzathine, Nursing, Primary Health Care, Drug-Related Side Effects and Adverse Reactions, Anti-Infective Agents., Penicilina G Benzatina, Enfermería, Atención Primaria de Salud, Efectos Colaterales y Reacciones Adversas Relacionados con Medicamentos, Antiinfecciosos.

## Abstract

**Objectives:**

to identify the nursing team’s perspective on Penicillin G Benzathine management in Primary Health Care.

**Methods:**

this is an online survey study with a quantitative approach, conducted with Primary Health Care nursing professionals from March to July 2023. Data were analyzed using descriptive statistics (mean, median, and standard deviation).

**Results:**

of the study participants, 75% had never witnessed an adverse event related to Penicillin G Benzathine administration, while the remainder reported only mild events. The main difficulties identified were concern about possible serious adverse events (54%), lack of standardization in collecting allergy history (40%), and uncertainty in administration (30%).

**Conclusions:**

the topics raised indicate that there are still uncertainties in Benzathine Penicillin management in Primary Health Care, making it essential to carry out educational actions to qualify nursing professionals’ knowledge and practice in this context.

## RESUMEN

**Objetivos:**

identificar la perspectiva del equipo de enfermería sobre el manejo de la Penicilina G Benzatínica en Atención Primaria de Salud.

**Métodos:**

estudio cuantitativo en línea, realizado con profesionales de enfermería de Atención Primaria de Salud entre marzo y julio de 2023. Los datos se analizaron mediante estadística descriptiva (media, mediana y desviación estándar).

**Resultados:**

de los participantes del estudio, el 75 % nunca había presenciado un evento adverso relacionado con la administración de Penicilina G Benzatínica, mientras que el resto solo reportó eventos leves. Las principales dificultades identificadas fueron preocupación por posibles eventos adversos graves (54%), falta de estandarización en la recopilación de antecedentes de alergias (40%) la incertidumbre en la administración (30%).

**Conclusiones:**

los temas abordados indican que aún existen incertidumbres en el manejo de la Penicilina Benzatínica en la Atención Primaria de Salud, siendo imprescindible la realización de acciones educativas para calificar el conocimiento y la práctica de los profesionales de enfermería en este contexto.

## INTRODUCTION

Despite high antimicrobial resistance and risks of serious adverse events, Penicillin G Benzathine is an extremely important and widely used drug, particularly in Primary Health Care (PHC), being one of the main entry points to the Brazilian Health System^(1)^.

It is indicated for treating different infections, and is essential for cases of syphilis, including in pregnant women^(2)^. It is the only drug that crosses the transplacental barrier and is capable of treating the fetus in utero, thus preventing congenital syphilis^(3)^.

However, there are still doubts among nursing professionals regarding the safety of its administration in PHC, due to possible serious adverse events, without the service having adequate infrastructure for this type of care^(1)^.

This situation may compromise the use of Penicillin G Benzathine in situations where it is recommended as the ideal drug^(4)^, leading to the unnecessary use of broader spectrum antimicrobial agents, which contributes to microbial resistance.

The Federal Nursing Council (In Portuguese, *Conselho Federal de Enfermagem* - COFEN) released a technical note in 2017 regulating Penicillin G Benzathine administration in PHC, including the need to establish protocols and flows for the early identification of suspected cases of anaphylaxis so that its use is safe^(5)^.

The most feared adverse event by professionals is the risk of death from anaphylaxis. To better understand this event and increase safety when administering Penicillin G Benzathine, it is essential to properly collect patients’ allergy history, and nurses are essential in this process^(6)^.

Little is known about the actual number of occurrences of these adverse events in Brazil. According to data from the Brazilian National Health Regulatory Agency, between January 2018 and early April 2023, 88 events involving the active ingredient Benzylpenicillin were reported. No deaths were reported during this period, and in 64% of cases, the outcome reported by the reporting party was recovery or resolution^(7)^. It is worth remembering that there is no way to estimate the underreporting of these events.

Considering the importance of this therapeutic option in PHC, as well as the need to expand scientific production that portrays the nursing team’s view on this practice and possible difficulties associated with this process, the present study was carried out.

## OBJECTIVES

To identify the nursing team’s view on Penicillin G Benzathine management in PHC.

## METHODS

### Ethical aspects

The project was approved by the *Universidade Federal de São Carlos* Research Ethics Committee, and data collection began. All participants signed an Informed Consent Form (ICF), and the study complied with all ethical requirements established by Resolution 466/2012.

Since data collection was conducted in a virtual environment, all requirements for research procedures involving any stage of the study were followed, in accordance with Circular Letter 01/2021/CONEP/SECNS/MoH and Law 13,709 of August 14, 2018, which regulates personal data protection. All data related to this study were stored on a personal computer, with an unshared login and password, and on physical media. No cloud-based records were maintained.

### Study design, period and location

This is descriptive survey research with a quantitative approach, conducted using an online questionnaire, prepared by the authors with the objective of identifying nursing professionals’ experience in Penicillin G Benzathine administration in PHC.

The questionnaire used via Google Forms^®^ was available to be answered from March to July 2023.

This study followed the recommendations proposed in the Checklist for Reporting Results of Internet E-Surveys for preparing and reporting the results of surveys conducted via the internet^(8)^.

### Population and inclusion criteria

Nursing assistants, nursing technicians, and nurses working in PHC from different regions of Brazil were included. Nursing professionals (assistants, technicians, and nurses) working in PHC who completed the instrument in full were excluded.

Due to the study design, a non-probabilistic sample was selected, consisting of professionals who responded to the invitation by completing the online questionnaire. The target population is difficult to pinpoint, as there may be multiple active registrations for the same professional. For educational purposes, the current population figures are as follows: 3.1 million nursing professionals, including 757,000 nurses, 1.9 million nursing technicians, and 475,000 nursing assistants registered with COFEN in 2024^(9)^.

### Study protocol

Data were collected through a questionnaire available on Google Forms^®^. Participants were recruited by distributing an invitation, including a link to the form, on social media (Facebook^®^, Instagram^®^, Telegram^®^, and LinkedIn^®^), targeting groups of primary care nursing professionals nationwide.

Information was disseminated on social media and social networks through the personal accounts of the authors and members of their research group, targeting specific groups of healthcare and nursing professionals, aiming to reach the target population. It was also disseminated on portals such as *FAPESP, Rádio UFSCar*, and *InfoRede UFSCar*, which offer nationwide access and viewing.

Those interested in participating in the study accessed the link available in the invitation, agreed to the ICF and responded to the instrument (survey).

The questionnaire used consists of an online survey instrument, composed of 27 closed-ended questions, developed by the authors and divided into three parts: A - With ten questions about the population sociodemographic profile and characterization of professional practices related to Penicillin G Benzathine (sex, race/color, age, workplace, length of professional experience, region of Brazil, professional category, length of professional training, education); B - With nine Likert-type questions, ranging from rarely to frequently, about the practice of administering Penicillin G Benzathine (muscle of application, safety in administration, frequency of use, indication, and population); and C - With eight multiple-choice questions about adverse events experienced and knowledge needs (type of events and outcomes; insecurity and barriers).

As this is an initial assessment of the nursing team’s perspective in this context, the instrument has not undergone validity. It was assessed by members of a research group, and a pilot test was conducted with five professionals. It is believed that the results of this study can then inform the development of new instruments and guide future studies.

### Analysis of results and statistics

The data were tabulated in a Microsoft Excel^®^ spreadsheet and analyzed using descriptive statistics, calculating position measures (mean and median) and dispersion measures (standard deviation), and constructing tables of absolute and relative frequencies.

## RESULTS

A total of 289 participants responded to the instrument, of which 152 (53%) were from the Southeast region, 73 (25%) from the Northeast region, 25 (12%) from the South, 19 (7%) from the North, and ten (3%) from the Central-West region. Of the total, 239 (83%) were women.

A total of 141 (49%) participants self-identified as white, 117 (40%) as brown, 25 (9%) as black, and five (2%) as yellow. One participant chose not to provide information. Regarding age, 112 (39%) participants were between 40 and 49 years old, with a mean of 42 years and a standard deviation of ten years.

Thus, 163 (56%) of respondents are nurses; 106 (37%) are nursing technicians; and 20 (7%) are nursing assistants. Concerning length of professional training, 177 (61%) of participants reported training time of more than ten years; 41 (14%) reported between six and ten years; 62 (22%) reported between one and five years; and nine (3%) reported less than one year.

In relation to time working in PHC, 111 (36%) of participants reported having worked for more than 10 years, 59 (20%) for six to ten years, 88 (31%) for one to five years, and 31 (11%) for less than one year.

Participants’ highest level of education varied according to their professional category. Among nurses, 66 (41%) had a specialization in PHC and related areas, while 36 (22%) had a specialization in other areas. Among nursing technicians, 74 (70%) had a technical degree as their highest level of education, and 21 (20%) had a bachelor’s degree. Similarly, among nursing assistants, nine (45%) had a technical degree as their highest level of education, and two (10%) had a bachelor’s degree.

Regarding section B of the questionnaire, referring to Penicillin G Benzathine administration, and the frequency of assessment of users’ allergy history, the results are shown in Table 1.

**Table 1:** Frequency of Benzylpenicillin administration and assessment of allergy history, Brazil, 2022

Action related to Benzylpenicillin	Frequency, n (%)				
	Always	Often	Sometimes	Rarely	Never
Benzylpenicillin administration	48 (17)	87 (30)	94 (32)	42 (15)	18 (6)
Assessment of users’ allergy history	177 (61)	17 (6)	19 (7)	37 (13)	39 (13)

Table 2 shows the data participants considered relevant during the process of investigating users’ allergy history. It is worth noting that more than one alternative could have been selected. It is noteworthy that 237 (82%) of participants considered it relevant to ask about previous allergic reactions during the allergy history investigation. In contrast, the need for hospitalization was indicated by 141 (49%) participants.

**Table 2:** Relevant information for assessing allergy history, Brazil, 2022

Relevant data in allergy history assessment - N = 289	
	**n (%)**
**Given**	
Previous allergic reactions	237 (82)
Reaction symptoms	220 (76)
Time for the event to manifest after using the medication	172 (59)
How long ago the reaction occurred	167 (58)
Medication suspected of triggering the allergic reaction	162 (56)
Need for hospitalization	141 (49)
Route of administration used	89 (31)

Regarding the most frequent indications for Benzetacil^®^ reported by participants, “syphilis” was the most frequently indicated, with 273 (94%) responses, followed by “tonsillitis” with 178 (62%), “rheumatic fever” with 133 (46%), “skin abscesses” with 119 (41%), and “erysipelas” with 102 (35%). Regarding the populations that most frequently receive Penicillin G Benzathine in PHC, adults obtained 268 (93%) of responses, followed by pregnant women, with 149 (52%). Both questions allowed more than one alternative to be selected by respondents.

The dorsum-gluteal region was indicated as the Penicillin G Benzathine administration site in 250 (86%) reports, followed by the ventrogluteal region with 71 (25%). The least used muscle for medication administration was the vastus lateralis of the thigh, with 35 (12%) responses. One respondent (0.35%) indicated the “deltoid” as the routine application site.

Table 3 describes the complications reported as having been experienced by participants during Penicillin G Benzathine preparation and administration. It is noteworthy that 184 (64%) participants indicated the lack of complete medication dilution as the most frequent complication during the drug preparation, and 244 (84%) indicated needle obstruction as the most frequent during Penicillin G Benzathine administration.

**Table 3:** Reported incidents during Penicillin G Benzathine preparation and administration, Brazil, 2022

Incidents experienced by participants, N = 289	
	**n (%)**
**Intercurrence during preparation**	
Medication not completely diluted	184 (64%)
Forgot to consider medication expansion in the final volume	29 (10%)
Broken vial	15 (5%)
**Intercurrence during administration**	
Needle clogged	244 (84%)
User worried	156 (54%)
User fainted	74 (26%)
User refused	67 (23%)
Wrong needle used	8 (3%)
Wrong site administered	1 (0.35%)

Concerning the availability of Penicillin G Benzathine in participants’ workplaces, 189 (65%) reported that the medication is always available; 63 (22%) stated that it is occasionally out of stock; 11 (4%) indicated that it is frequently out of stock; and 26 (9%) reported that the medication is not dispensed in their workplace.

Regarding part C of the questionnaire, 72 (25%) participants reported having witnessed adverse events after medication administration. Table 4 presents the adverse events witnessed. Again, more than one alternative could be selected. The percentages presented considered only participants who witnessed events (N=72).

**Table 4:** Adverse events witnessed by participants after Penicillin G Benzathine administration, Brazil, 2022

Adverse events witnessed after Penicillin G Benzathine administration, N = 72^*^	
	**n (%)**
**Event**	
Fainting	55 (76%)
Hives and other skin manifestations	27 (37%)
Diffuse pruritus	15 (21%)
Glottic edema	9 (12%)
Seizure	7 (10%)
Anaphylactic shock	5 (7%)
Cardiopulmonary arrest	1 (1%)

* *It is allowed to select more than one alternative.*

The measures necessary to manage the incident, reported by the 72 participants who witnessed adverse events, were: emergency care at the unit itself by 45 (62%) participants; suspension of treatment by 18 (25%) participants; continuation of treatment by 17 (24%) participants; hospitalization and emergency care by the Mobile Emergency Care Service, indicated by eight (11%) participants.

Twenty-two (70%) participants reported feeling safe Penicillin G Benzathine administration in PHC. On the other hand, 157 (54%) stated that they did not feel safe dealing with possible adverse reactions resulting from medication administration.

The topic of adverse events associated with Penicillin G Benzathine administration was addressed during the academic training of 186 (64%) participants. The lack of in-service training on drug administration was highlighted by 240 (83%) participants. Finally, 151 (52%) indicated knowledge of Resolution 3 of 2017 of COFEN, which discusses Penicillin G Benzathine administration in PHC.

## DISCUSSION

The 289 professionals participating in the study, from the five regions of Brazil, with a predominance of the Southeast region (152; 53%) and women (239; 83%), are aligned with the profile of the profession in Brazil, according to COFEN^(5)^.

In the present study, nurses accounted for 53% of participants, whereas, in reality, they correspond to approximately 23% of professionals that make up the nursing team, according to COFEN^(5)^. This disparity may be attributed to nurses’ greater familiarity with research.

As for training time and professional experience, it is clear that most participants (218; 75.4%) completed their training more than six years ago and that more than half of respondents (170; 58.8%) have worked in PHC for more than six years. Professional experience plays a fundamental role in improving PHC care quality. In fact, the literature points to a positive correlation between years of experience and nursing professional performance^(10)^.

Caring for pregnant women with syphilis is one of the activities that can have a positive impact on professionals’ experience. This includes everything from identifying the case through rapid testing to reporting, prescribing, and administering treatment as well as providing guidance about the disease^(11)^. It is important to note that, although curable, syphilis has seen an increase in incidence in recent years. Due to its long incubation period and complex clinical manifestations, it is often difficult to diagnose. However, Penicillin G Benzathine remains the first-line treatment for all stages of the disease^(12)^.

In research carried out in England, from 2014 to 2021, Penicillin G Benzathine was one of the most prescribed and dispensed medicines by non-medical professionals, such as nurses^(13)^. This practice highlights the crucial role of nursing in ensuring the population’s access to the indicated treatment, in particular Penicillin G Benzathine.

An essential factor in Penicillin G Benzathine administration, including in PHC, is collecting an allergy history. Although, in this study, more than half of nurses reported collecting this information as part of medical history, it is clear that there is no clarity or standardization regarding which items should be investigated, including by other members of the nursing team.

In this context, the categorization and standardization of nursing diagnoses proposed by the NANDA-I document, in its 12^th^ edition, defines an allergic reaction as “susceptible to an exaggerated immune response or reaction to substances, which may compromise health”. Diagnosing allergy risk is part of the Safety and Security domain. NANDA-I addresses the importance of documenting allergy history, which can contribute to standardization of diagnosis and patient safety^(14)^.

Although correctly identifying allergy history directly impacts patient safety, the national patient safety program and other regulations do not specify how this process should occur. Basic patient safety protocols include ensuring safety in the prescription, use, and medication administration, with an emphasis on the need to record allergies in medical records and prescriptions. However, they do not describe how this process should occur, implying a lack of standardization of this activity in healthcare services^(15-17)^.

Regarding international initiatives to assess a patient’s allergy history, there is no consensus on this. However, several authors point to topics that should be considered in this process, such as event description, suspected triggering medication, route of administration, time elapsed since event onset, length of time since the potential event occurred, necessary management (medication discontinuation, hospitalization, use of antihistamines, among others), prior use of suspected medication, and re-exposure or use of another medication in the same class after the suspected event^(18,19)^.

In a study conducted in the United States, nurses expressed uncertainty about determining whether the episode reported by patients was, in fact, an allergy to Penicillin G Benzathine. Barriers to history collection included a high workload, lack of physical space and equipment, lack of training to identify and manage allergies at the PHC level, lack of presence at the time of the event for clinical evaluation, and a shortage of nursing staff^(20)^.

Penicillin G Benzathine allergy assessment is not considered routine in PHC, not only in Brazil^(21,22)^. In the United States, about 15% of patients report an allergy to Penicillin G Benzathine, when in fact, upon further analysis, less than 1% actually have an allergy^(6)^. Inadequate classification regarding the real risk of allergy to Penicillin G Benzathine may imply the shift from the first line of treatment to a second line, often with a broader spectrum, which results in an increase in adverse events, antimicrobial resistance, and increased costs^(2,21,23,24)^.

Problems such as incomplete dilution and needle obstruction mentioned are consistent with the literature, since the drug has extremely low solubility^(25)^. Furthermore, factors related to medication quality, including the raw material used for its preparation and good manufacturing practices, can also influence the final quality, potentially leading to incomplete dilution. The already diluted form can help minimize these problems; however, it is expensive and heat-sensitive, requiring adaptation for storage and transportation. It is also unavailable in many countries^(26)^. Therefore, following the precise recommendations for reconstitution, dilution and use of appropriate ingredients in preparation can minimize the problems presented.

Most respondents had never experienced any adverse event after taking Penicillin G Benzathine. It is important to note that true anaphylactic reactions to Penicillin G Benzathine are rare, occurring in approximately 0.01% of cases, with vasovagal reactions being more common^(25)^. Furthermore, lethality associated with immediate hypersensitivity reactions triggered by the use of beta-lactam antimicrobial agents, such as Penicillin G Benzathine, is 0.001%^(27)^.

The current clinical protocol and therapeutic guideline for Comprehensive Care for People with Sexually Transmitted Infections points out that anaphylaxis with the use of Benzylpenicillin is not exclusive to this class of medication, and the fear of adverse events should not be an impediment to its use, especially in PHC^(28)^. This conduct is supported by COFEN Resolution 3 of 2017, which almost half of participants are not familiar with, although it is an important instrument that clarifies and legitimizes the actions surrounding Penicillin G Benzathine administration by the nursing team^(5)^.

In this study, most of the events witnessed included fainting and hives/other skin manifestations, supporting international literature^(2,29)^, and correspond to events of possible intervention in the PHC itself.

It is worth noting that most professionals report never having participated in courses or training on the topic in the workplace, and others reported not even participating during their training, highlighting a pressing need for in-service education. This situation contributes to insecurity and often results in refusal to administer this antimicrobial in PHC^(20,23)^.

### Study limitations

One limitation of this study is that participants were selected from a convenience sample, without a sample size calculation, and this should be considered when interpreting the findings presented here. Furthermore, the study design incorporates recall bias, a common bias in surveys and interviews, as it relies on participant self-reporting.

### Contributions to health, nursing or public policy

It is expected that the data evidenced in this research will support new studies and even educational actions so that Penicillin G Benzathine can be used safely in PHC, equipping the nursing team with the appropriate information on allergy history collection and the management of possible adverse events.

## CONCLUSIONS

This study allowed us to identify nursing perspectives on Penicillin G Benzathine administration, highlighting common complications in the preparation and administration of this medication in PHC, such as difficulty in diluting the drug and needle obstruction during administration. A lack of uniformity in the allergy history collection process was observed as well as difficulty in prioritizing the items to be collected. Regarding adverse events resulting from Benzylpenicillin use, a small portion of participants experienced some event in their practice, with fainting and skin manifestations being the most prevalent, while serious events were rare.

It is concluded that the topics raised indicate that there are still uncertainties in Benzathine Penicillin in PHC management, and that it is essential to carry out educational actions in order to qualify nursing professionals’ knowledge and practice in this context.

## Data Availability

The research data are available only upon request.

## References

[1] Araújo TCV, Souza MB. (2020). Adesão das equipes aos testes rápidos no pré-natal e administração da penicilina benzatina na atenção primária. Rev Esc Enferm USP.

[2] Gartlan WA, Rahman S, Reti K., StatPearls (2023). Benzathine Penicillin.

[3] Domingues CSB, Duarte G, Passos MRL, Sztajnbok DCN, Menezes MLB. (2021). Protocolo Brasileiro para Infecções Sexualmente Transmissíveis 2020: sífilis congênita e criança exposta à sífilis. Epidemiol Serv Saúde.

[4] Silva RCL, Peregrino AAF, Rocco R, Ribeiro LR, Machado DA, Silva CRL. (2022). Cost utility of penicillin use in primary care for the prevention of complications associated with syphilis. J Bras Doenças Sex Transm.

[5] Conselho Federal de Enfermagem (Cofen) (2017). Nota Técnica COFEN/CTLN/No 03/2017.

[6] Jishna Shrestha A, Zahra F, Cannady P, Affiliations J. (2023). Antimicrobial Stewardship.

[7] Agência Nacional de Vigilância Sanitária (Anvisa) (2022). Notificações de Farmacovigilância.

[8] Eysenbach G. (2004). Improving the Quality of Web Surveys: the Checklist for Reporting Results of Internet E-Surveys (CHERRIES). J Med Internet Res.

[9] Conselho Federal de Enfermagem (Cofen) (2024). Quantitativo de profissional por regional.

[10] Landu ZK, Crowley T. (2023). Primary health care nurses’ knowledge, self-efficacy and performance of diabetes self-management support. Afr J Prim Health Care Fam Med.

[11] Barimacker SV, Zocche DAA, Zanatta AE, Rodrigues JD, Korb A. (2022). Construção de fluxograma e protocolo de enfermagem para manejo da sífilis na Atenção Primária em Saúde. Ciênc Cuid Saúde.

[12] Sadoghi B, Stary G, Wolf P. (2023). Syphilis. JDDG.

[13] Courtenay M, Gillespie D, Lim R. (2023). Patterns of GP and nurse independent prescriber prescriptions for antibiotics dispensed in the community in England: a retrospective analysis. J Antimicrobial Chemother.

[14] Herdman TH, Takao C, Kamitsuru S. (2021). NANDA International Nursing Diagnoses: definitions and classification 2021-2023.

[15] Agência Nacional de Vigilância Sanitária (Anvisa) (2013). Resolução da diretoria colegiada - RDC no 36, de 25 de julho de 2013.

[16] Agência Nacional de Vigilância Sanitária (Anvisa) (2013). Portaria no 529, de 01 de abril de 2013.

[17] Agência Nacional de Vigilância Sanitária (Anvisa) (2013). Portaria no 1.377, de 09 de julho de 2013.

[18] Shenoy ES, Macy E, Rowe T, Blumenthal KG. (2019). Evaluation and management of penicillin allergy: a review. JAMA.

[19] Carter EJ, Schramm C, Baron K, Zolla MM, Zavez K, Banach DB. (2023). Perceived usefulness of a mnemonic to improve nurses’ evaluation of reported penicillin allergies. Antimicrob Steward Healthc Epidemiol.

[20] Alagoz E, Saucke M, Balasubramanian P, Lata P, Liebenstein T, Kakumanu S. (2023). Barriers to penicillin allergy de-labeling in the inpatient and outpatient settings: a qualitative study. Allergy, Asthma, and Clin Immunol.

[21] Wurcel AG, Guardado R, Ortiz C, Bornmann CR, Gillis J, Huang K (2022). Low frequency of allergy referral for penicillin allergy evaluation in an urban Boston primary care setting. J Allergy Clin Imunol.

[22] Patrick DM, Al Mamun A, Smith N, Rempel E, Calissi P, Blondel-Hill E (2019). Beta-lactam allergy: benefits of de-labeling can be achieved safely. BCMJ.

[23] Bediako H, Dutcher L, Rao A, Sigafus K, Harker C, Hamilton KW (2022). Impact of an inpatient nurse-initiated penicillin allergy delabeling questionnaire. Antimicrob Steward Healthc Epidemiol.

[24] Blumenthal KG, Peter JG, Trubiano JA, Phillips EJ. (2019). Antibiotic allergy. Lancet.

[25] Oliveira JSA, Melo FA, Vieira CENK, Costa EO, Ribeiro ABTS, Nascimento JL, Silva MLO (2019). Cuidado seguro na administração de Penicilina G Benzatina em crianças com febre reumática: relato de experiência. Rev Soc Bras Enferm Ped.

[26] Hand RM, Senarathna SMDKG, Page-Sharp M, Gray K, Sika-Paotonu D, Sheel M (2020). Quality of benzathine penicillin G: A multinational cross-sectional study. Pharmacol Res Perspect.

[27] Fica A, Muñoz D, Rojas T, Sanzana C, Muñoz C. (2020). Penicillin desensitization in allergic pregnant women with syphilis: report of two cases. Rev Med Chile.

[28] Ministério da Saúde (BR) (2021). Resolução nº 12, de 19 de abril de 2021.

[29] Yip DW, Gerriets V. (2022). Penicillin: continuing education activity.

