# Supplementary material for: Penicillin G Benzathine management in Primary Health Care: nursing’s perspective
Source: Rev Bras Enferm. 2026 May 11;79:e20240385. doi: 10.1590/0034-7167-2024-0385 (PMC13169624; doi:10.1590/0034-7167-2024-0385)
Supplement: Translation

# Translation: Manejo da Penicilina G Benzatina na Atenção Primária à Saúde: a visão da enfermagem

**DOI:** 10.1590/0034-7167-2024-0385pt

**Authors:** Giovanna Martins Biondi, Daniela Sanches Couto, Lívia Cristina Scalon da Costa Perinoti, Tatiane Garcia do Carmo Flausino, Ana Joice de Barros Figueiredo, Rosely Moralez de Figueiredo

**Affiliations:** I Universidade Federal de São Carlos. São Carlos, São Paulo, Brasil; II Centro Universitário das Faculdades Associadas de Ensino. São João da Boa Vista, São Paulo, Brasil; III Universidade Federal de São Carlos, Hospital Universitário. São Carlos, São Paulo, Brasil; IV Universidade de Araraquara. Araraquara, São Paulo, Brasil

**Keywords:** Penicilina G Benzatina, Enfermagem, Atenção Primária à, Saúde, Efeitos Colaterais e Reações Adversas Relacionados a Medicamentos, Anti-Infecciosos.

## Abstract

**Objetivos:**

identificar a visão da equipe de enfermagem sobre o manejo da Penicilina G Benzatina na Atenção Primária à Saúde.

**Métodos:**

estudo do tipo *survey online*, de abordagem quantitativa, realizado com profissionais de enfermagem da Atenção Primária à Saúde de março a julho de 2023. Os dados foram analisados por estatística descritiva (média e mediana e desvio padrão).

**Resultados:**

dos participantes do estudo, 75% nunca presenciaram um evento adverso relacionado à administração da Penicilina G Benzatina, enquanto os demais relataram apenas eventos leves. As principais dificuldades identificadas foram preocupação com possíveis eventos adversos graves (54%), falta de padronização na coleta do histórico de alergias (40%) e insegurança na administração (30%).

**Conclusões:**

os temas levantados indicam que ainda existem incertezas no manejo da Penicilina Benzatina na Atenção Primária à Saúde, sendo essencial a realização de ações educativas para qualificar o conhecimento e a prática do profissional de enfermagem nesse contexto.

## INTRODUÇÃO

Apesar da resistência antimicrobiana elevada e de riscos de eventos adversos graves, a Penicilina G. Benzatina é um medicamento de extrema importância e muito utilizado, particularmente, na Atenção Primária à Saúde (APS), sendo uma das principais portas de entrada para o Sistema Único de Saúde^(1)^.

É indicada para o tratamento de diferentes infecções, sendo essencial para os casos de sífilis, inclusive em gestantes^(2)^. É o único medicamento que atravessa a barreira transplacentária e é capaz de tratar o feto intraútero, evitando, assim, a sífilis congênita^(3)^.

Entretanto, ainda há dúvidas, por parte dos profissionais de enfermagem, quanto à segurança da sua administração na APS, devido a possíveis eventos adversos graves, sem que haja no serviço infraestrutura adequada para esse tipo de atendimento^(1)^.

Essa situação pode comprometer o uso da Penicilina G. Benzatina em situações onde é preconizada como a droga ideal^(4)^, induzindo à utilização desnecessária de antimicrobianos de maior espectro, o que contribui para resistência microbiana.

O Conselho Federal de Enfermagem (COFEN) divulgou em 2017 uma nota técnica regulamentando a administração da Penicilina G Benzatina na APS, incluindo a necessidade de se estabelecer protocolos e fluxos de identificação precoce de casos suspeitos de anafilaxia, de forma que seu uso seja seguro^(5)^.

O evento adverso mais temido pelos profissionais é o risco de morte por anafilaxia. Visando uma melhor compreensão desse evento e de aumento da segurança na realização da administração da Penicilina G Benzatina, é fundamental a coleta adequada do histórico de alergias do paciente, e o enfermeiro é imprescindível nesse processo^(6)^.

Pouco se sabe sobre os reais números de ocorrências desses eventos adversos no país. Segundo dados da Agência Nacional de Vigilância Sanitária, entre janeiro de 2018 e o início de abril de 2023, foram notificados 88 eventos envolvendo o princípio ativo Benzilpenicilina. Nenhum óbito foi relatado no período, e em 64% dos casos, o desfecho apontado pelo notificador foi de recuperado ou resolvido^(7)^. Cabe lembrar que não há como estimar a subnotificação desses eventos.

Considerando a importância dessa opção terapêutica na APS, bem como a necessidade de ampliar a produção científica que retrate a visão da equipe de enfermagem sobre essa prática e possíveis dificuldades associadas a esse processo, realizou-se o presente estudo.

## OBJETIVOS

Identificar a visão da equipe de enfermagem sobre o manejo da Penicilina G Benzatina na APS.

## MÉTODOS

### Aspectos éticos

O projeto foi aprovado pelo Comitê de Ética em Pesquisa da Universidade Federal de São Carlos e, após, iniciada a coleta de dados. Todos os participantes registraram anuência no Termo de Consentimento Livre e Esclarecido (TCLE), e o estudo respeitou todos os aspectos éticos estabelecidos pela Resolução n^o^ 466/2012.

Como se tratou de coleta de dados em ambiente virtual, foram seguidas todas as exigências para procedimentos em pesquisas com qualquer etapa em ambiente virtual, de acordo com a Carta Circular n^o^ 01/2021/CONEP/SECNS/MS e com a Lei nº 13.709, de 14 de agosto de 2018, que dispõe sobre a proteção de dados pessoais. Todos os dados referentes a esta pesquisa foram armazenados em computador pessoal, com *login* e senha não compartilhados e em mídia física. Não foram mantidos registros em nuvem.

### Desenho, período e local do estudo

Pesquisa descritiva, do tipo *survey*, com abordagem quantitativa, conduzida utilizando questionário *online*, elaborado pelas autoras com o objetivo de identificar a experiência dos profissionais de enfermagem na administração da Penicilina G Benzatina na APS.

O questionário via *Google Forms* ^®^ utilizado ficou disponível para ser respondido no período de março a julho de 2023.

O presente estudo seguiu as recomendações propostas no *checklist* Checklist for Reporting Results of Internet *E-Surveys* para elaboração e relato de resultados de pesquisas realizadas via internet^(8)^.

### População e critérios de inclusão

A população do estudo foi composta por auxiliares, técnicos de enfermagem e enfermeiros atuantes na APS, de diferentes regiões do Brasil. Como critérios de inclusão foram selecionados profissionais de enfermagem (auxiliares, técnicos e enfermeiros), atuantes na APS, que responderam ao instrumento integralmente.

Devido ao desenho do estudo, foi determinada uma amostra não probabilística, formada pelos profissionais que atenderam o convite respondendo ao questionário *online.* A população-alvo é de difícil precisão, uma vez que pode haver múltiplos registros ativos de um mesmo profissional. Para fins didáticos, seguem os números atuais da população: 3,1 milhões de profissionais de enfermagem, sendo 757 mil enfermeiros, 1,9 milhões técnicos de enfermagem e 475 mil auxiliares de enfermagem registrados no COFEN no ano de 2024^(9)^.

### Protocolo do estudo

Os dados foram coletados por meio de questionário, disponibilizado na plataforma *Google Forms^®^*. O recrutamento dos participantes ocorreu por divulgação de convite, com o *link* de acesso ao formulário, nas redes e mídias sociais (*Facebook^®^, Instagram^®^, Telegram^®^* e *Linkedin^®^*), com foco em grupos formados por profissionais de enfermagem da APS de todo o território nacional.

A divulgação feita nas redes e mídias sociais foi realizada por meio das contas pessoais das autoras e integrantes do grupo de pesquisa no qual estão inseridas, direcionada para grupos específicos de profissionais de saúde e de enfermagem, almejando atingir a população-alvo. Também foi realizada divulgação em portais como FAPESP, Rádio UFSCar e InfoRede UFSCar, de acesso e visualização nacional.

Aqueles que se interessaram em participar do estudo acessaram o *link* disponível no convite divulgado, concordaram com o TCLE e responderam ao instrumento (*survey*).

O questionário utilizado consiste em um instrumento do tipo *survey online*, composto por 27 questões fechadas, elaborado pelos autores e dividido em três partes: A - Com dez questões acerca do perfil sociodemográfico da população e caracterização das práticas profissionais relacionadas à Penicilina G Benzatina (sexo, raça/cor, idade, local de trabalho, tempo de atuação profissional, região do Brasil, categoria profissional, tempo de formação profissional, escolaridade); B - Com nove questões tipo Likert, variando de raramente a frequentemente sobre a prática de administração da Penicilina G Benzatina (músculo de aplicação, segurança na administração frequência de uso, indicação e população); e C - Com oito questões de múltipla escolha sobre eventos adversos vivenciados e necessidades de conhecimento (tipo de eventos e de desfechos; insegurança e barreiras).

Por se tratar de diagnóstico inicial da visão da equipe de enfermagem nesse contexto, o instrumento não passou por validação. Ele foi avaliado por integrantes de grupo de pesquisa, sendo realizado teste piloto com cinco profissionais. Acredita-se que os resultados deste estudo possam então subsidiar a elaboração de novos instrumentos e direcionar estudos futuros.

### Análise dos resultados e estatística

Os dados foram tabulados em planilha no *Microsoft Excel* ^®^ e analisados com estatística descritiva, calculando-se medidas de posição (média e mediana) e medidas de dispersão (desvio padrão), e construindo-se tabelas de frequências absolutas e relativas.

## RESULTADOS

Responderam ao instrumento um total de 289 participantes, sendo 152 (53%) provenientes da região Sudeste, 73 (25%), da região Nordeste, 25 (12%), da Sul, 19 (7%), da Norte, e dez (3%), da região Centro-Oeste. Do total, 239 (83%) eram mulheres.

Autodeclararam-se pertencentes à raça/cor branca 141 (49%) dos participantes, parda, 117 (40%), preta, 25 (9%), e amarela, cinco (2%). Um participante preferiu não informar. Com relação à idade, 112 (39%) participantes estão entre 40-49 anos, com média de 42 anos e desvio padrão de dez anos.

São enfermeiros 163 (56%) dos respondentes, 106 (37%) técnicos de enfermagem e 20 (7%) auxiliares de enfermagem. No que se diz respeito ao tempo de formação profissional, 177 (61%) dos participantes referiram tempo de formação superior a dez anos, enquanto 41 (14%), entre seis e dez anos, 62 (22%), entre um e cinco anos, e nove (3%), menos de um ano.

Quanto ao tempo de trabalho na APS, 111 (36%) dos participantes relataram possuir mais de 10 anos, 59 (20%), de seis a dez anos, 88 (31%) de um a cinco anos, e 31 (11%), menos de um ano.

A maior escolaridade dos participantes variou de acordo com sua categoria profissional. entre os enfermeiros, 66 (41%) possuem especialização na APS e áreas correlatas, enquanto 36 (22%) têm especialização em outras áreas. Entre os técnicos de enfermagem, 74 (70%) possuem o técnico como maior escolaridade e 21 (20%) possuem graduação. Similarmente, entre os auxiliares de enfermagem, nove (45%) possuem o técnico como maior escolaridade, e dois (10%) possuem graduação.

Em relação à seção B do questionário, referente à administração da Penicilina G Benzatina, e à frequência de avaliação do histórico de alergias de usuários, os resultados se encontram dispostos na Tabela 1.

**Tabela 1:** Frequência da administração de Benzilpenicilina e avaliação do histórico de alergia, Brasil, 2022

Ação relacionada à Benzilpenicilina	Frequência, n (%)				
	Sempre	Muitas vezes	Às vezes	Raramente	Nunca
Administração de Benzilpenicilina	48 (17)	87 (30)	94 (32)	42 (15)	18 (6)
Avaliação do histórico de alergia do usuário	177 (61)	17 (6)	19 (7)	37 (13)	39 (13)

A Tabela 2 demonstra os dados considerados relevantes pelos participantes durante o processo de investigação do histórico de alergia dos usuários. É válido pontuar que mais de uma alternativa poderia ser selecionada. Destaca-se que 237 (82%) dos participantes consideram relevante questionar a respeito de reações alérgicas prévias durante a investigação do histórico de alergia; em contrapartida, a necessidade de hospitalização é apontada por 141 (49%) participantes.

**Table 2:** Relevant information for assessing allergy history, Brazil, 2022

Dados relevantes na avaliação do histórico de alergia - N = 289	
	**n (%)**
**Dado**	
Reações alérgicas prévias	237 (82)
Sintomas da reação apresentada	220 (76)
Tempo para manifestação do evento após o uso do medicamento	172 (59)
Há quanto tempo ocorreu a reação	167 (58)
Medicamento suspeito de desencadear a reação alérgica	162 (56)
Necessidade de hospitalização	141 (49)
Via de administração utilizada	89 (31)

Quanto às indicações mais frequentes da Benzetacil^®^ relatadas pelos participantes, “sífilis” foi a mais apontada, com 273 (94%) respostas, seguida de “amigdalite”, com 178 (62%), “febre reumática”, com 133 (46%), “abscessos cutâneos”, com 119 (41%), e “erisipela” com 102 (35%). Quanto às populações que mais frequentemente recebem a Penicilina G Benzatina na APS, adultos obtiveram 268 (93%) das respostas, seguidos das gestantes, com 149 (52%). Ambas as questões permitiam que mais de uma alternativa fosse selecionada pelos respondentes.

A região do dorso glúteo foi apontada como o local de administração da Penicilina G Benzatina em 250 (86%) sinalizações, seguida da ventroglúteo, com 71 (25%). O músculo menos utilizado para aplicação do medicamento foi o vasto lateral da coxa, com 35 (12%) das respostas. Um respondente (0,35%) assinalou o “deltóide” como o local de aplicação rotineiro.

A Tabelas 3 descreve as intercorrências relatadas como já vivenciadas pelos participantes durante o preparo e administração da Penicilina G Benzatina. Destaca-se que 184 (64%) participantes apontam a falta de diluição completa do medicamento como intercorrência mais frequente durante o preparo do fármaco, e 244 (84%) apontam a obstrução da agulha como a mais frequente durante a administração da Penicilina G Benzatina.

**Tabela 3:** Intercorrências relatadas como ocorridas apontadas durante o preparo e administração da Penicilina G Benzatina, Brasil, 2022

Intercorrências vivenciadas pelos participantes, N = 289	
	**n (%)**
**Intercorrência durante o preparo**	
Medicamento não diluiu completamente	184 (64%)
Esqueceu de considerar expansão do medicamento no volume final	29 (10%)
Ampola quebrou	15 (5%)
**Intercorrência durante a administração**	
Agulha entupiu	244 (84%)
Usuário receoso	156 (54%)
Usuário desmaiou	74 (26%)
Recusa do usuário	67 (23%)
Utilização da agulha errada	8 (3%)
Administração no local errado	1 (0,35%)

Em relação à disponibilidade de Penicilina G Benzatina nos locais de trabalho dos participantes, 189 (65%) relataram que sempre há o medicamento disponível; 63 (22%) afirmam eventualmente estar em falta; 11 (4%) assinalaram que frequentemente está em falta; e 26 (9%) relatam não haver dispensação do medicamento em seu local de trabalho.

Quanto à parte C do questionário, 72 (25%) participantes referiram já ter presenciado eventos adversos após a administração do medicamento. A Tabela 4 apresenta os eventos adversos presenciados. Novamente, mais de uma alternativa poderia ser assinalada. Os percentuais apresentados consideraram apenas os participantes que presenciaram eventos (N=72).

**Tabela 4:** Eventos adversos presenciados pelos participantes após administração da Penicilina G Benzatina, Brasil, 2022

Eventos adversos presenciados após administração da Penicilina G Benzatina, N = 72^*^	
	**n (%)**
**Evento**	
Desmaio	55 (76%)
Urticária e outras manifestações cutâneas	27 (37%)
Prurido difuso	15 (21%)
Edema de glote	9 (12%)
Convulsão	7 (10%)
Choque anafilático	5 (7%)
Parada cardiorrespiratória	1 (1%)

* *Permitido assinalar mais de uma alternativa.*

As medidas necessárias para condução do ocorrido referidas pelos 72 participantes, que presenciaram eventos adversos, foram: atendimento de urgência na própria unidade, por 45 (62%) participantes; suspensão do tratamento, por 18 (25%) participantes; continuidade do tratamento, por 17 (24%) participantes; hospitalização e atendimento de urgência pelo Serviço de Atendimento Móvel de Urgência, apontados por oito (11%) participantes.

Referiram se sentir seguros para administrar Penicilina G Benzatina na APS 202 (70%) dos participantes. Por outro lado, 157 (54%) afirmaram não sentir segurança em lidar com eventuais reações adversas decorrentes da administração do medicamento.

O tema eventos adversos associados à administração da Penicilina G Benzatina foi abordado durante a formação acadêmica de 186 (64%) participantes. Já a ausência de treinamentos em serviço sobre a administração do medicamento foi apontada por 240 (83%) participantes. Por fim, 151 (52%) sinalizaram conhecer a Resolução nº 3 de 2017 do COFEN, que discorre sobre a administração da Penicilina G Benzatina na APS.

## DISCUSSÃO

Os 289 profissionais participantes do estudo, oriundos das cinco regiões do Brasil, com predominância da região Sudeste (152; 53%) e de mulheres (239; 83%), estão alinhados com o perfil da profissão no Brasil, segundo o COFEN^(5)^.

No presente estudo, os enfermeiros corresponderam a 53% dos participantes, sendo que, na realidade, correspondem a cerca de 23% dos profissionais que compõem a equipe de enfermagem, segundo o COFEN^(5)^. Essa disparidade pode ser atribuída à maior familiaridade dos enfermeiros com a pesquisa.

Quanto ao tempo de formação e experiência profissional, evidencia-se que a maioria dos participantes (218; 75,4%) concluiu sua formação há mais de seis anos e que mais da metade dos respondentes (170; 58,8%) atua há mais de seis anos na APS. A experiência profissional desempenha um papel fundamental na melhoria da qualidade das ações realizadas na APS. De fato, é apontada na literatura uma correlação positiva entre os anos de experiência e o desempenho do profissional de enfermagem^(10)^.

O atendimento a gestantes com sífilis é uma das atividades que pode sofrer impacto positivo com relação à experiência do profissional. Isso inclui desde a identificação do caso por meio do teste rápido até a notificação, prescrição e administração do tratamento, proporcionando também orientações acerca da doença^(11)^. É importante destacar que, embora curável, a sífilis apresenta um aumento da incidência nos últimos anos. Por apresentar um longo período de incubação e manifestação clínica complexa, muitas vezes, é de difícil diagnóstico. Contudo, a Penicilina G Benzatina continua sendo a primeira escolha para todos os estágios da doença^(12)^.

Em pesquisa realizada na Inglaterra, de 2014 a 2021, a Penicilina G Benzatina foi um dos medicamentos mais prescritos e dispensados por profissionais não médicos, como o enfermeiro^(13)^. Essa prática ressalta o papel crucial da enfermagem em garantir o acesso da população ao tratamento indicado, em particular da Penicilina G Benzatina.

Um fator imprescindível para administração da Penicilina G Benzatina, inclusive na APS, é a coleta do histórico de alergia. Embora, na presente pesquisa, mais da metade dos enfermeiros tenha relatado que o coletam, como parte da anamnese, nota-se que não há clareza e padronização sobre quais itens devem ser investigados, inclusive pelos demais componentes da equipe de enfermagem.

Nesse contexto, a categorização e padronização de diagnósticos em enfermagem proposto pelo documento NANDA-I, em sua 12ª edição, define a reação alérgica como “suscetível a uma resposta imune exagerada ou reação a substâncias, que pode comprometer a saúde”. O diagnóstico de risco de alergia faz parte do domínio Segurança e Proteção. A NANDA-I aborda a importância de documentar o histórico de alergia, podendo contribuir para padronização do diagnóstico e para segurança do paciente^(14)^.

Embora a correta identificação do histórico de alergia impacte diretamente a segurança do paciente, o programa nacional de segurança do paciente e demais normativas não apontam como esse processo deve ocorrer. Entre os protocolos básicos de segurança do paciente, está prevista a segurança na prescrição, uso e administração dos medicamentos, com destaque para necessidade do registro de alergia em prontuário e prescrição, sem, contudo, descrever como esse processo deve ocorrer, implicando ausência de padronização dessa atividade nos serviços de saúde^(15-17)^.

Quanto às iniciativas internacionais para avaliação do histórico de alergia do paciente, não há um consenso a respeito dessa atividade. Entretanto, diversos autores apontam tópicos que devem ser considerados neste processo, tais como descrição do evento, medicamento suspeito de desencadear o evento, a via de administração, o tempo transcorrido para o início do evento, há quanto tempo o potencial evento ocorreu, o manejo necessário (suspensão do medicamento, hospitalização, uso de anti-histamínicos, entre outros), uso prévio do medicamento suspeito, e reexposição ou uso de outro medicamento da mesma classe após o evento suspeito^(18,19)^.

Em estudo conduzido nos Estados Unidos, enfermeiros expressaram insegurança ao determinar se o episódio relatado pelo paciente foi, de fato, uma alergia à Penicilina G Benzatina. Barreiras para coleta do histórico incluíram uma alta carga de trabalho, falta de espaço físico e equipamentos, além de ausência de treinamento para identificar e manejar alergias em nível de APS, não estar presente no momento do evento para avaliação clínica e escassez de profissionais de enfermagem^(20)^.

A avaliação da alergia à Penicilina G Benzatina não é considerada algo rotineiro na APS não somente no Brasil^(21,22)^. Nos Estados Unidos, cerca de 15% dos pacientes relatam alergia à Penicilina G Benzatina, quando, na verdade, após uma análise mais aprofundada, menos de 1% realmente apresentam alergia^(6)^. A classificação inadequada quanto ao risco real de alergia à Penicilina G Benzatina pode implicar o deslocamento da primeira linha de tratamento para uma segunda linha, muitas vezes de maior espectro, o que resulta no aumento de eventos adversos, resistência antimicrobiana e aumento de custos^(2,21,23,24)^.

Os problemas como diluição incompleta e obstrução da agulha mencionados são compatíveis com a literatura, uma vez que o medicamento possui solubilidade extremamente baixa^(25)^. Além disso, fatores relacionados à qualidade do medicamento, incluindo a matéria-prima para o seu preparo e as boas práticas de fabricação, também podem influenciar a qualidade final, podendo desencadear uma diluição incompleta. A apresentação já diluída pode contribuir para minimizar esses problemas, entretanto, possui custo elevado e é termossensível, necessitando de adequação para seu armazenamento e transporte, e não está disponível em muitos países^(26)^. Dessa forma, seguir as recomendações precisas na reconstituição, diluição e uso de insumos adequados no preparo pode minimizar os problemas apresentados.

A maioria dos respondentes nunca presenciou algum tipo de evento adverso após a administração da Penicilina G Benzatina. É importante destacar que a ocorrência de reações anafiláticas verdadeiras à Penicilina G Benzatina é rara, ocorrendo em cerca de 0,01% dos casos, sendo mais comuns as reações vasovagais^(25)^. Além disso, a letalidade associada a reações de hipersensibilidade imediata desencadeada pelo uso de antimicrobianos betalactâmicos, como a Penicilina G Benzatina, é de 0,001%^(27)^.

O atual protocolo clínico e diretriz terapêutica para Atenção Integral às Pessoas com Infecção Sexualmente Transmissível pontua que a anafilaxia com uso de Benzilpenicilina não é exclusiva desta classe de medicamento, e o receio da ocorrência de eventos adversos não deve ser um impeditivo para o seu uso, especialmente na APS^(28)^. Esta conduta é corroborada pela Resolução nº 3 de 2017 do COFEN, a qual quase metade dos participantes não está familiarizado, embora seja um importante instrumento que esclarece e legitima as ações em torno da administração da Penicilina G Benzatina pela equipe de enfermagem^(5)^.

Neste estudo, a maior parte dos eventos presenciados incluiu desmaio e urticária/outras manifestações de pele, corroborando a literatura internacional^(2,29)^, e correspondem a eventos de possível intervenção na própria APS.

Vale ressaltar que a maioria dos profissionais menciona nunca ter participado de cursos ou treinamentos sobre o tema no local de trabalho, e outros referiram ainda que não participaram nem durante sua formação, expondo uma premente necessidade de educação em serviço. Esse panorama contribui para insegurança e resulta, muitas vezes, na recusa da administração desse antimicrobiano na APS^(20,23)^.

### Limitações do estudo

Como limitações deste estudo, pontua-se o fato de que a seleção dos participantes foi realizada a partir de uma amostra por conveniência, sem cálculo amostral, e que deve ser considerada na interpretação dos achados aqui apresentados. Ademais, o desenho do estudo incorpora o viés de memória, um viés comumente presente em inquéritos e entrevistas, visto depender do relato do participante.

### Contribuições para a área da saúde, enfermagem ou políticas públicas

Espera-se que os dados evidenciados nesta pesquisa subsidiem novos estudos e até mesmo ações educativas para que a Penicilina G Benzatina seja utilizada na APS de maneira segura, instrumentalizando a equipe de enfermagem quanto à coleta adequada do histórico de alergias e ao manejo de possíveis eventos adversos.

## CONCLUSÕES

Este estudo permitiu identificar a visão da enfermagem na administração da Penicilina G Benzatina, destacando-se intercorrências comuns no preparo e administração desse medicamento na APS, como a dificuldade na diluição do fármaco e obstrução da agulha durante a administração. Foi observada ausência de uniformidade no processo de coleta de histórico de alergia, bem como dificuldade em priorizar os itens que devem ser coletados. Quanto aos eventos adversos decorrentes do uso da Benzilpenicilina, uma parcela pequena dos participantes vivenciou em sua prática algum evento, sendo os mais prevalentes desmaio e manifestações cutâneas, e raros, os eventos graves.

Conclui-se que os temas levantados indicam que ainda existem incertezas no manejo da Penicilina Benzatina na APS, sendo essencial a realização de ações educativas a fim de qualificar o conhecimento e a prática do profissional de enfermagem neste contexto.

## Data Availability

Os dados de pesquisa só estão disponíveis mediante solicitação.
